# Can we swallow the idea of azathioprine as the next treatment option for pediatric eosinophilic esophagitis?

**DOI:** 10.1186/1939-4551-8-S1-A11

**Published:** 2015-04-08

**Authors:** Priyanka Lall

**Affiliations:** 1 Guthrie Medical Group, USA

## Background

Eosinophilic esophagitis (EoE) is a burgeoning health concern impacting a growing proportion of the population. Experts advise utilization of either dietary modifications, swallowing of topical steroids or a combination for management. We present a child with EoE refractory to diet and topical corticosteroids who improved with oral azathioprine (AZA).

## Methods

We have a case of a 6 year old boy diagnosed with EOE after presenting with dysphagia to solids more so than liquids associated with severe odynophagia and vomiting. While on a PPI, the esophageal biopsy results revealed micro-abscesses, a thickened squamous basal layer and >150 Eos/hpf with normal esophageal pH monitoring. There was no evidence of increased eosinophils in the gastric and duodenal biopsies and no peripheral blood eosinphilia. Skin prick testing was negative to a panel of 70 foods including cow’s milk. Food patch testing revealed no evidence of delayed hypersensitivity. He failed to respond to several months swallowed topical fluticasone propionate or swallowed topical budesonide. A trial of ciclesonide swallowed was initiated but his clinical symptoms persisted. Due to his prior failure with swallowed topical corticosteroids, prednisone 5mg every other day was started in addition to budesonide 1mg inhalation suspension. During follow-up one month later, he had worsening symptoms consisting of increased vomiting, dysphagia with soft foods and generalized abdominal pain. The prednisone dose was discontinued and he was started on AZA.

## Results

Follow-up four months later revealed significant improvement in his clinical symptoms. He had no symptoms of solid food dysphagia, odynophagia, impaction, abdominal pain or vomiting. His follow-up EGD still demonstrated hyperplastic basal cell layer but a tenfold reduction in intraepithelial eosinophils with a peak count of 15 Eos/hpf in his esophagus and no other histologic findings consistent with EoE. 18 months after starting AZA, his peak esophageal biopsy intraepithelial count remained at 15 Eos/hpf and his duodenum and stomach remained normal. To date he continues to tolerate AZA and has not manifested any drug adverse side effects. His growth has actually improved while on AZA.

## Conclusions

AZA has previously been used as monotherapy to induce long term remission in adults with refractory corticosteroid-dependent EoE. Current review of the literature yielded no prior reports of safely using AZA in the pediatric EoE population to successfully reduce clinical symptoms and tissue eosinophilia as observed in our case. This index case report suggests that AZA can be considered as a safe adjuvant therapy in recalcitrant pediatric EoE cases.

